# Adsorptive Removal of Azo Dye New Coccine Using High-Performance Adsorbent-Based Polycation-Modified Nano-Alpha Alumina Particles

**DOI:** 10.1155/2022/9425334

**Published:** 2022-02-07

**Authors:** Thi Hai Yen Doan, Hong Anh Pham, Ngoc Huyen Nguyen, Thi Dung Le, Thanh Binh Nguyen, Thanh Son Le

**Affiliations:** ^1^Faculty of Chemistry, University of Science, Vietnam National University, Hanoi-19 Le Thanh Tong, Hoan Kiem, Hanoi, Vietnam; ^2^Nguyen Sieu High School, Yen Hoa, Cau Giay, Hanoi, Vietnam

## Abstract

The azo dyes new coccine (NCC) were successfully removed through the adsorption onto PVBTAC-modified *α*-Al_2_O_3_ particles. The optimal conditions of both the surface modification by PVBTAC adsorption and the NCC adsorption were thoroughly investigated. Formerly, polycations PVBTAC were adsorbed onto the nanosized *α*-Al_2_O_3_ particles at *pH* 8, NaCl 100 mM, with a contact time of 2 h, and initial concentration of 1000 ppm to modify the *α*-Al_2_O_3_ surface. Latterly, the NCC adsorptive removal was conducted at *pH* 8, NaCl 10 mM, *α*-Al_2_O_3_ adsorbent dosage of 3 mg mL^−1^, and a contact time of 45 min. Interestingly, the optimal *pH* of 8 potentially applies to treat real wastewater as the environmental *pH* range is often about 7–8. High removal efficiency and adsorption capacity of the NCC azo dyes were, respectively, found to be approximately 95% and 3.17 mg g^−1^ with an initial NCC concentration of 10 ppm. The NCC adsorption on the modified *α*-Al_2_O_3_ particles was well fitted with a Freundlich model isotherm. A pseudo-second kinetic was more suitable for the NCC adsorption on the PVBTAC-modified *α*-Al_2_O_3_ surface than a pseudo-first kinetic. The NCC adsorptive removal kinetic was also affirmed by the FT-IR spectra, based especially on the changes of functional group stretch vibrations of −SO_3_^−^ group in the NCC molecules and −N^+^(CH_3_)_3_ group in the PVBTAC molecules. The high reusability of the *α*-Al_2_O_3_ particles was proved to be higher than 50% after four generation times.

## 1. Introduction

Nowadays, the azo dyes have been diversely used in many industrial applications such as textile, clothing, printing, plastic, and paper productions. The azo dyes that contain a specific group −*N* = *N*− are the most popular and estimate to be about 50–70% of annual total dye production [1]. The azo dyes durably exist and are non-degraded in the aquatic environment. Moreover, the effluents from these industries often contain a large dye amount of 10–200 mg L^−1^ [2, 3], which is likely dangerous to the ecosystem and human health, causing biohazard risks such as bioaccumulation and mutagenesis inducing genetical diseases, congenital malformations, or cancer [4–6]. For this reason, the dye elimination from the wastewater is necessary.

The most common dye treatment techniques basing on discoloration are physical, chemical, biological methods, and adsorption [7–12]. For decades, a lot of research on the azo dye demand removal from aqueous solution has been conducted [10, 13]. Among them, new coccine (NCC), well known with another name as Acid Red 18, has been recently taken the interest of scientists. NCC is one of the synthetic organic azo dyes widely used in textile industries. NCC degradation by using the TiO_2_ photocatalyst in combination with H_2_O_2_ in a cavitation reactor was intensified up to 88.1% in comparison to 64.8% obtained in an ultrasonic reactor. NCC degraded up to 99.6% with the employment of the Fenton reagent combining with UV irradiation (UV/FR) at *pH* 3 and under 30 min of reaction time [14]. Among these techniques, adsorption is more effective with high removal efficiency, and simple and low cost. The use of carbon as an adsorbent seems to be popular. NCC was totally removed (higher than 99% in 10 consecutive cycles) from an aqueous solution through adsorbing on carbon nanotubes coated with chitosan at a high temperature of 323.15 K [15]. A maximum NCC removal efficiency reached 90.83% by adsorption onto the almond shell-synthesized activated charcoal at the optimal *pH* 2 [16]. It was found that the use of carbon nanotubes with multiple walls (MWCNTs) could remove 166.67 mg g^−1^ of the NCC from an aqueous solution at *pH* 3 [17]. On the other hand, other materials are also applied as adsorbents. NCC was removed from wastewater by means of adsorbing onto the powder of zero iron at an acidic condition of *pH* 3 [18]. However, strictly controlled conditions, such as high experimental temperature or/and acidic environment, have been requested as the removal efficiency is still low.

Furthermore, alumina has been well known as a potential adsorbent for organic pollutant treatment. Alpha alumina (*α*-Al_2_O_3_) was characterized with a high specific surface area and existed as the most stable form at high temperature among various alumina forms such as beta (*β*)- and gamma (*γ*)- [19–21]. Recently, the adsorptive removal potential of the organic pollutants was intensified through modifying the *α*-Al_2_O_3_ surface by polyelectrolyte adsorption, even at room temperature, which was previously proved [22–24]. It was found that the sodium dodecyl sulfate (SDS) surfactant was strongly attached to the alpha alumina of a small specific surface area [23]. Continuously, oxytetracycline antibiotic was adsorptively removed with a high removal efficiency of higher than 90% by using SDS-modified *α*-Al_2_O_3_ [24]. The modification of surface materials has been demonstrated to increase the total net surface charges, enhancing adsorptive removal of wastewater pollutants through electrostatic interactions [25–28]. The adsorption mechanism of the azo dyes were followed Langmuir [15, 29], Freundlich [15, 30], and two-step models [31], while adsorption kinetics were indicated by first-order and second-order models [18, 32, 33].

In this study, attention was focused on removing NCC via adsorption onto polycation-modified *α*-Al_2_O_3_ nanosized particles under different conditions at room temperature. The effectiveness parameters including pH, ionic strength, adsorbent dosage, contact time, and the initial concentration on both *α*-Al_2_O_3_ modification process and the NCC removal were comprehensively investigated. The adsorption mechanism was discussed based on both the FT-IR spectra and the general adsorption isotherm models such as Langmuir, Freundlich and two-step. Adsorption kinetics were described by the pseudo-first and -second models.

## 2. Materials and Methods

### 2.1. Materials

Nanosized alpha alumina (*α*-Al_2_O_3_) particles solvothermaly synthesized following the previous method, were used as adsorbents [34]. Accordingly, to precipitate alumina hydroxide, 4 M sodium hydroxide solution was added to 1 M aluminum nitrate solution, respectively prepared from NaOH pellets and (Al(NO_3_)_3_ (analytical reagent, Merk, Germany). The precipitates were dried at 80°C for 24 h after centrifugation and rising to neutral *pH* with ultrapure water. Then, the collected precipitates were transformed to *α*-Al_2_O_3_ at high temperature of 1200°C for 12 h. Finally, the *α*-Al_2_O_3_ were dried and ground after activating by using the solution of 0.05 M NaOH and rising with ultrapure water several times. A high synthesis yield of the *α*-Al_2_O_3_ particles was calculated to be approximately 97.23 ± 1.43%. The *α*-Al_2_O_3_ particles synthesized were nanosized at about 27 nm determined by transmission electron microscopy (TEM, H7650, Hitachi, Tokyo, Japan). Homopolymer, poly(vinylbenzyl) trimethylammonium chloride) (PVBTAC) with a molecular weight of 343.45 g mol^−1^ (Hyogo, Japan) was applied as a surface modifier.

**Figure 1:**
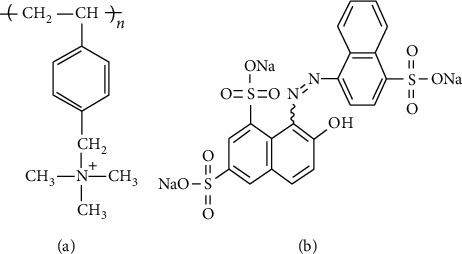
Chemical structures of (a) PVBTAC and (b) NCC.

The stock PVBTAC solution of 10^4^ ppm was prepared for adsorption experiments. New coccine with a molecular weight of 604.46 g mol^−1^ (NCC, purity >82% Merck, Germany) was used as azo dye. The polymer working solutions were diluted from the stock solution. The chemical structures of polycation PVBTAC and the NCC dye were described in Figure 1. The NaCl solutions of 0.1 and 1 M (prepared from analytical reagent NaCl, Merk, Germany) were employed to control ionic strength after filtering through cellulose membranes of 0.2 *μ*m pore size. Meanwhile, the solutions of 0.1 M HCl and 0.1 M NaOH (Merk, Germany) were used to adjust the *pH* of the solution under a pH meter (Hanna, USA). All experimental solutions were prepared with ultrapure water (resistance of 18.2 MΩ cm, Labconco, Kansai, MO, USA).

### 2.2. Modification of Alpha-Alumina Using Highly Positively Charged Polycation

#### 2.2.1. Alpha-alumina Modification by the PVBTAC Adsorption

The nanosized *α*-Al_2_O_3_ particles were strongly shaken for 2 h by an orbital shaker before using. To deaggregate particles, the nanosized *α*-Al_2_O_3_ particles were sonicated for 30 min before conducting each experimental modification. To modify the particles, suitable PVBTAC stock solution volumes were added to the nanosized *α*-Al_2_O_3_ particles. The modification experiments were carried out for about 2 h by vigorously shaking in investigating conditions of pH and ionic strength. Then, the samples were centrifuged to separate the solid-liquid phases. Finally, the solutions were collected to determine PVBTAC-remaining concentration by ultraviolet-visible (UV-Vis) measurement.

#### 2.2.2. NCC Adsorptive Removal Using PVBTAC-Modified Nanosized *α*-Al2O3 Particles

The nanosized *α*-Al_2_O_3_ particles modified by PVBTAC adsorption at optimal conditions were rinsed with ultrapure water to eliminate excess polycation PVBTAC after centrifuging to release remaining water. Then, these modified materials were used to conduct dye removal experiments at room temperature and different conditional parameters such as *pH*, ionic strength, adsorption time, *α*-Al_2_O_3_ adsorbent, and NCC adsorbate dosage. Similarly, the solution was pipetted after centrifugation of the sample. The NCC concentration remaining in the solution was measured by the UV-Vis method. Each experiment was at least triply repeated. Standard deviations were determined by at least triple experiments.

### 2.3. Methods

#### 2.3.1. Ultraviolet Visible (UV-Vis) Spectroscopy

The PVBTAC and NCC concentrations were determined by an UV-Vis spectroscopy equipped with a spectrophotometer (UV-1650 PC, Shimadzu, Japan) at a wavelength of 224 and 508 nm, respectively.

The PVBTAC adsorption efficiency and the NCC removal efficiency (*H*, %) were determined

(1) $$\begin{array}{c} H=\frac{C_{i}-C_{e}}{C_{i}}\times 100\%. \end{array}$$

where *C*_*i*_ and C_*e*_ are initial and equilibrium polymer concentrations (ppm), respectively.

The polymer adsorption capacities onto unmodified/modified nanosized *α*-Al_2_O_3_ particles were determined by equation (2):

(2) $$\begin{array}{c} \Gamma =\frac{C_{i}-\;C_{e}}{m}\;\times M\times 1000, \end{array}$$

where Γ is the polymer adsorption capacity (mg g^−1^) at contact time *t* (min), *M* is polymer molecular weight (g mol^−1^), and *m* is the *α*-Al_2_O_3_ adsorbent dosage (mg mL^−1^).

#### 2.3.2. Adsorption Mechanism

The adsorption isotherms of PVBTAC onto the *α*-Al_2_O_3_ particles and NCC onto the PVBTAC-modified *α*-Al_2_O_3_ particles were considered to be fit with some general adsorption isotherm models such as Langmuir, Freundlich, and two-step [26]. Each adsorption isotherm model was described as below.

First, the Langmuir model described by equation (3) was applicable for absorbate-formed monolayer on the absorbent. The adsorption favorite was evaluated by *R*_*L*_ constant as in equation (4).

(3) $$\begin{array}{c} \frac{C_{e}}{Г}=\frac{1}{K_{L}\cdot {Г}_{\max}}+\frac{C_{e}}{{Г}_{\max}}, \end{array}$$

(4) $$\begin{array}{c} R_{L}=\frac{1}{1+K_{L}\cdot C_{i}}. \end{array}$$

where *K*_*L*_ is Langmuir constant.

Second, the concept of the Freundlich model was developed, based on the experimental data, to evaluate that multiple adsorbate layer formed on the inhomogeneous adsorbent surface. It was subjected in

(5) $$\begin{array}{c} Г=K_{f}\cdot C_{e}^{n}, \end{array}$$

where *K*_*f*_ is Freundlich constant and *n* is the layer number.

A general equation of the two-step model adsorption isotherm [34] is as follows:

(6) $$\begin{array}{c} \Gamma =\frac{\Gamma_{\max}k_{1}C_{e}\left(\left(1/n\right)+k_{2}C_{e}^{n-1}\right)}{1+k_{1}C_{e}\left(1+k_{2}C_{e}^{n-1}\right)}, \end{array}$$

where *k*_1_ and *k*_2_ are equilibrium constants for in the first and second adsorption step, respectively.

#### 2.3.3. Adsorption Kinetics

Adsorption kinetics of polymer are often described by the pseudo-first and pseudo-second models proposed by Lagergren as follows [17, 26]:

(7) $$\begin{array}{c} \ln\left({Г}_{e}-Г\right)=\ln\;{Г}_{e}-K_{1}t,\\ \frac{t}{Г}=\frac{1}{K_{2}\cdot {Г}_{e}^{2}}+\frac{1}{{Г}_{e}}t, \end{array}$$

where *Г* is polymer adsorption capacity at contact time *t* (mg g^−1^), Γ_*e*_ is the polymer adsorption capacity (mg g^−1^) at equilibrium state, and *K*_1_ (min^−1^) and *K*_2_ (g mg^−1^ min^−1^) are reaction rate constants of the pseudo-first and pseudo-second models, respectively.

#### 2.3.4. Fourier Transform Infrared (FT-IR) Spectroscopy

The mechanisms of adsorption of both PVBTAC onto the *α*-Al_2_O_3_ particles and NCC onto the PVBTAC-modified *α*-Al_2_O_3_ particles were discussed based on the FT-IR spectra. The PVBTAC adsorption and the NCC adsorption were carried out for 2 h at pH 8 and at ionic strength of NaCl 100 and 10 mM, respectively. Then, the residuals were collected and dried at 80°C after centrifugation and removal of the water excess. Five spectra of the *α*-Al_2_O_3_ particles, PVBTAC, NCC, PVBTAC-modified *α*-Al_2_O_3_ particles, and NCC-adsorbed-PVBTAC-modified *α*-Al_2_O_3_ particles were recorded from 400 to 4000 cm^−1^ by an Affinity-1S spectrophotometer (20 scans averaging, Shimadzu, Japan) at room temperature (293 K).

## 3. Results and Discussions

### 3.1. Modification through PVBTAC Adsorption on the Synthesized Nanosized *α*-Al_2_O_3_ Particles

#### 3.1.1. *pH* Effect

The solution *pH* significantly affected the PVBTAC adsorption onto the synthesized nanosized *α*-Al_2_O_3_ particles. To modify the *α*-Al_2_O_3_ particles, an initial PVBTAC concentration of 50 ppm was added to the particles of 5 mg mL^−1^ in the *pH* range of 4–12 under different ionic strength conditions of 1, 10, and 100 mM NaCl.

**Figure 2:**
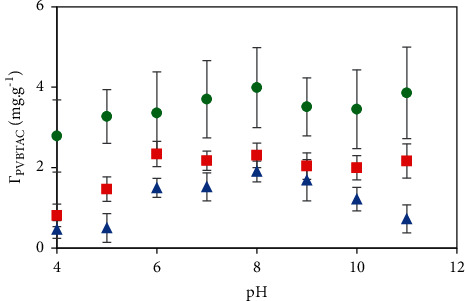
*pH* effect on the PVBTAC adsorption on the *α*-Al_2_O_3_ particles in different ionic strengths: (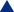) 1 mM, (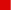) 10 mM and (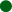) 100 mM NaCl with *C*_*i*,PVBTAC_ of 50 ppm, t of 2 h and *m*_*α*-Al2O3_ of 5 mg mL^−1^.

Figure 2 shows that PVBTAC adsorption capacity (*Г*_PVBTAC_) increased with increasing *pH* until *pH* reached 8, then *Г*_PVBTAC_ decreased with continuous increment of the *pH*. The *α*-Al_2_O_3_ particles were characterized with −O and OH functional groups [19] and an isoelectric point (IEP) of approximately 6.7 [35]. It means that the charging sign of the *α*-Al_2_O_3_ particles changed over the IEP point. At the *pH* higher than 6.7, the *α*-Al_2_O_3_ particle surface was negatively charged due to the presence of O^−^ groups while PVBTAC was positively charged, independently from the *pH* level. Therefore, the PVBTAC adsorption onto the *α*-Al_2_O_3_ particles was promoted due to electrostatic attractions at the solution *pH* greater than 6.7. Oppositely, at a *pH* lower than 6.7, the *α*-Al_2_O_3_ particle surface was positively charged due to appearance of the OH_2_^+^ groups. These surface groups introduced the strong electrostatic repulsions between PVBTAC molecules and the *α*-Al_2_O_3_ surface, resulting in the limitation of the PVBTAC adsorption. At the solution pH of 8, the Г_PVBTAC_ achieved maximum value at all ionic strengths. Hence, *pH* 8 was chosen to be the optimal modification condition.

#### 3.1.2. Ionic Strength Effect

In addition to the pH effect, the ionic strength is one of the most effective parameters that influences to the adsorption capacity. The electrolyte shielding effect prevents the hydrophilic interactions and promote the hydrophobic interactions [36–38]. The *Г*_PVBTAC_ on the *α*-Al_2_O_3_ particles was determined at the four NaCl concentrations of 1, 10, 100 and 150 mM at *pH* 8, PVBTAC initial concentration of 50 ppm, contact time of 2 h and a *α*-Al_2_O_3_ adsorbent dosage of 5 mg mL^−1^.

**Figure 3:**
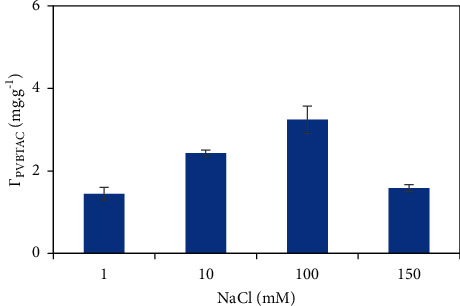
Ionic strength effect on the PVBTAC adsorption on the *α*-Al_2_O_3_ particles at *pH* 8, *C*_*i*,PVBTAC_ of 50 ppm, t of 2 h and *m*_*α*-Al2O3_ of 5 mg mL^−1^.

As obviously seen in Figure 3, the *Г*_PVBTAC_ increased with increasing the NaCl concentration from 1 to 100 mM and decreases with continuous salt increment. It was subjected to contributions of different interaction kinds on the PVBTAC adsorption such as electrostatic interactions, including electrostatic attraction and electrostatic repulsion, and non-electrostatic interactions such as hydrogen bonding and Van der Waals. The Van der Waals not only between PVBTAC and the *α*-Al_2_O_3_ particles, but also between PVBTAC molecules, might take responsibility for the *Г*_PVBTAC_ increment as the NaCl concentration went up from 1 to 100 mM. On the other hand, the electrolyte ions screened the electrostatic attraction between PVBTAC and the absorbent, the *Г*_PVBTAC_ quickly dropped while the NaCl concentration passed 100 mM. A maximum *Г*_PVBTAC_ of 3.24 mg g^−1^ was obtained at NaCl 100 mM. Hence, the ionic strength of 100 mM NaCl was employed to modify the *α*-Al_2_O_3_ surface.

#### 3.1.3. PVBTAC Initial Concentration Effect

**Figure 4:**
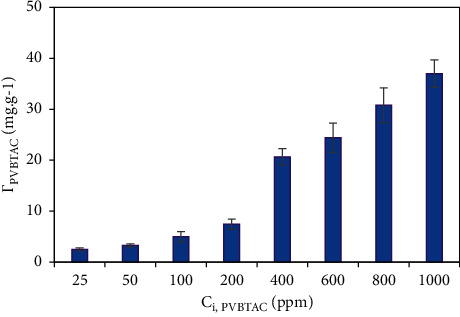
Initial concentration effect on the PVBTAC adsorption on the *α*-Al_2_O_3_ particles at pH 8, NaCl 100 mM, t of 2 h and *m*_*α*-Al2O3_ of 5 mg mL^−1^.

The polycation initial concentration effect on the *α*-Al_2_O_3_ modification efficiency was examined with the PVBTAC initial concentration from 25 to 1000 ppm at *pH* 8, NaCl 100 mM, contact time of 2 h. As shown in Figure 4, the *Г*_PVBTAC_ considerably raised from 2.46 to 37 mg g^−1^ by increasing the PVBTAC initial concentration in the range of 25–1000 ppm. The results can infer that more PVBTAC molecules were diffused and attached to the *α*-Al_2_O_3_ surface, due to main electrostatic interactions at high PVBTAC initial concentration and vice versa [38]. For the next experiments, the PVBTAC initial concentration of 1000 ppm was used to sufficiently modify the *α*-Al_2_O_3_ surfaces.

Summarily, the *α*-Al_2_O_3_ particle modification through PVBTAC adsorption was optimized at *pH* 8, ionic strength of 100 mM, contact time of 2 h, and PVBTAC initial concentration of 1000 ppm.

### 3.2. NCC Adsorptive Removal by Using PVBTAC-Modified *α*-Al_2_O_3_ Particles Confirmed by FT-IR Measurement

**Figure 5:**
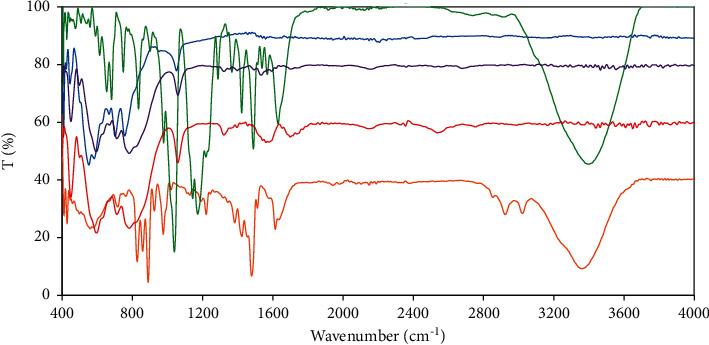
FT-IR spectra of: (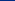) *α*-Al2O3 particles, (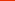) polycation PVBTAC, (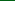) azo dye NCC, (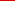) PVBTAC-modified *α*-Al2O3 particles and (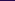) NCC-adsorbed-PVBTAC-modified *α*-Al2O3 particles.

The successful *α*-Al_2_O_3_ surface modification by adsorbing polycation PVBTAC, and the NCC dye adsorptive removal through adsorption onto the PVBTAC-modified particles were confirmed based on the changes of functional groups determined by FT-IR spectroscopy. Formerly, the PVBTAC adsorption onto the *α*-Al_2_O_3_ particles was affirmed by comparing the FT-IR spectra of the pure *α*-Al_2_O_3_ particles and the pure PVBTAC with the PVBTAC-modified *α*-Al_2_O_3_ particle spectrum (Figure 5). The Al-O vibration of the *α*-Al_2_O_3_ was attributed at 446, 552, 582, 702, and 756 cm^−1^ [39] while a sharp peak of Al-OH vibration was obtained at 1051 cm^−1^ [40]. At the investigated pH 8 and NaCl 100 mM, O^−^, O, and OH groups existed on the *α*-Al_2_O_3_ surface while only −N^+^*R*_3_ specified for PVBTAC (R abbreviating for −CH_3_). Hence, the functional group vibrations will change if the interactions happen. The sharp peak at 1051 cm^−1^ presenting for the Al-OH vibration of the *α*-Al_2_O_3_ moved to 1059 cm^−1^ in the PVBTAC-modified *α*-Al_2_O_3_ spectrum. Besides, R_3_N^+^-C vibration arose at 1853, 2924, 3050, and 3360 cm^−1^ and especially, a strong band at 976 cm^−1^ assigned to C-N^+^*R*_3_ in the PVBTAC spectrum, disappeared in the PVBTAC-modified *α*-Al_2_O_3_ particle spectrum [41, 42]. These changes recognize the electrostatic attractions between amide in the PVBATC molecule and O^−^ the *α*-Al_2_O_3_.

Lately, the *S* = *O* vibration at 1173 and 1144 cm^−1^ and the symmetrical −SO_3_^−^ stretching vibration at 1040 cm^−1^ of the NCC molecule disappeared in the PVBTAC-modified *α*-Al_2_O_3_ spectrum [43–45]. In addition to that, the R_3_N^+^-C vibration at 1800–3400 cm^−1^ and C-N peak at 976 cm^−1^ of PVBTAC in the spectrum of NCC-adsorbed-PVBTAC-modified *α*-Al_2_O_3_ [41, 42] were absent, strongly confirming the electrostatic attractions between −SO_3_^−^ of NCC and −N^+^*R*_3_ of PVBTAC. At the optimal *pH* 8 and NaCl 10 mM, the −OH group in the NCC was partly transferred to OH_2_^+^ as the pK_a_ is 11.38 [46], reducing its total negative charges as well as the electrostatic repulsion between the NCC molecules. We might imply that hydrogen bonding was formed among –OH groups between the NCC molecules because the peak at 3399 cm^−1^ corresponding to −OH vibration in the NCC molecule, disappeared in the spectrum of NCC-adsorbed-PVBTAC-modified *α*-Al_2_O_3_ [16, 44].

### 3.3. NCC Removal by Using the PVBTAC-modified *α*-Al_2_O_3_ Particles

#### 3.3.1. Ionic Strength Effect

**Figure 6:**
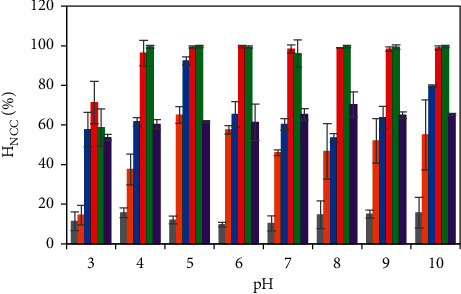
Ionic strength effect on the NCC adsorption onto the *α*-Al_2_O_3_ particles: without PVBTAC modification at (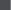) 10 mM NaCl and with PVBTAC modification at: (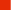) 0, (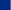) 1, (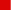) 10, (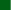) 100 and (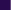) 200 mM NaCl. Other conditions are *C*_i, NCC_ of 10 ppm, *t* of 2 h, and *m*_*α*-Al2O3_ of 5 mg mL^−1^.

The removal efficiency of 10 ppm initial NCC concentration was more significantly enhanced by using the PVBTAC-modified *α*-Al_2_O_3_ adsorbents compared with using the unmodified nanosized *α*-Al_2_O_3_ particles at the same ionic strength of 10 mM NaCl and all *pH* (Figure 6). The modification of the *α*-Al_2_O_3_ adsorbents by PVBTAC adsorption improved the surface net charge, inducing additional electrostatic attractions between the positively charged PVBTAC covered on the *α*-Al_2_O_3_ surfaces and the negatively charged NCC molecules. Following this, the common effect factors to optimize the NCC removal through adsorbing onto the PVBTAC-modified *α*-Al_2_O_3_ adsorbents were comprehensively investigated.

As represented in Figure 6, two trends of the ionic strength effect on the NCC removal efficiency were observed. First, the ionic strength increment from 0 to 10 mM NaCl impulsed the NCC removal efficiency through adsorption onto the PVBTAC-modified *α*-Al_2_O_3_ particles at all *pH*. It can be explained that the promotion of the hydrogen bonding, and limitation of electrostatic repulsion between the NCC molecules, due to the condenser presence of electrolyte ions mainly controlled the NCC adsorption. However, the NCC removal efficiency decreased as the salt concentration was higher than 100 mM NaCl. The phenomenon suggested the significant shielding electrolyte ion effect on screening the electrostatic attractions between −N^+^*R*_3_ of the PVBTAC and −SO_3_^−^ of the dye NCC. It was consistent with our previous findings [36–38]. Herein, the NCC removal efficiency strongly depended on the interaction types between the NCC molecules and the PVBTAC-modified *α*-Al_2_O_3_ surface. Therefore, in all experiments, the ionic strength was controlled at NaCl 10 mM because the removal efficiency of the NCC was up to approximately 96% at almost all pH, and the standard deviation was lowest.

#### 3.3.2. *pH* Effect

**Figure 7:**
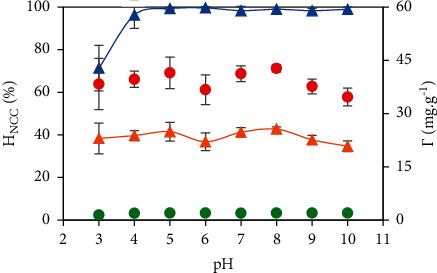
*pH* effect on the NCC adsorption onto the PVBTAC-modified *α*-Al_2_O_3_ particles at *pH* 8, NaCl 10 mM, *t* of 2 h and different conditions as *C*_*i*,NCC_ of 10 ppm, *m*_*α*-Al2O3_ of 3 mg mL^−1^: (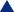) *H*_NCC_ (blue grid line) and (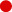) *Г*_NCC_ (points); and *C*_*i*,NCC_ of 100 ppm, *m*_*α*-Al2O3_ of 1 mg mL^−1^: (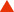) *H*_NCC_ (orange grid line) and (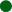) Г_NCC_ (points).

As could be seen in Figure 7, the NC removal efficiency, *H*_NCC_ and the adsorption amount, *Г*_NCC_ unremarkably changed with the wide change of *pH* from 3 to 12. PVBTAC is highly positively charged and independent on *pH*. Normally, the −OH group of the NCC molecule more becomes −OH_2_^+^ in lower *pH*, reducing the NCC net negative charge. As a result, the electrostatic attraction between PVBCTAC and NCC is less strong at low *pH* than at high *pH*. However, as observed, the OH_2_^+^ formation contribution was negligible at pH lower than pK_a_ of NCC (pK_a_ 11.38) [46]. It is suggested that the NCC net charge was mainly decided by the sulfate groups. On the other hand, at the same salt concentration, the *Г*_NCC_ got maximum at *pH* 8. The NCC removal was carried out at *pH* 8 and 10 mM NaCl.

#### 3.3.3. Absorbent Dosage Effect

**Figure 8:**
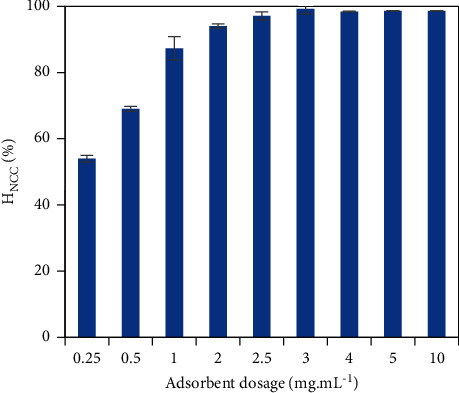
Effects of absorbent dosage on the NCC adsorption onto the PVBTAC-modified *α*-Al_2_O_3_ particles with *C*_*i*,NCC_ of 10 ppm, *pH* 8, NaCl 10 mM, *t* of 2 h, and *m*_*α*-Al2O3_ of 3 mg mL^−1^.

Normally, the total specific area significantly rises with an increment of absorbent dosage, enhancing more effectively adsorptive removal. The removal efficiency gradually changed from 53.94% to 99.25% with a 12-fold increment of the *α*-Al_2_O_3_ adsorbent dosage from 0.25 to 3 mg mL^−1^ (Figure 8). Then it was noticeably unchanged at the adsorbent dosage over 3 mg mL^−1^. Herein, the adsorbent amount of 3 mg mL^−1^ was sufficient for the adsorptive removal of NCC.

#### 3.3.4. Contact Time Effect

**Figure 9:**
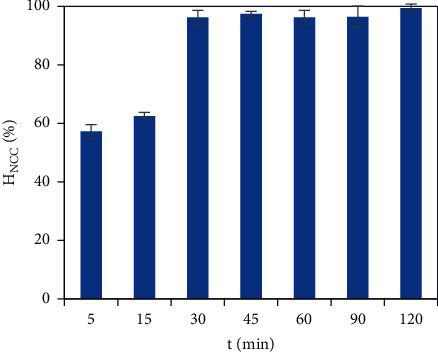
Effects of the absorbate dosage on the NCC adsorption onto the PVBTAC-modified *α*-Al_2_O_3_ particles with *C*_*i*,NCC_ of 10 ppm, *pH* 8, NaCl 10 mM and *m*_*α*-Al2O3_ of 3 mg mL^−1^.

The contact time effect on the NCC removal efficiency can be clearly observed in Figure 9. The NCC removal efficiency raised rapidly in each 15 min of the first 30 min. In the contact time range of 30–45 min, the removal efficiency continuously slowly raised from 96.25 to 97.40%. Then the removal efficiency was kept constant at the high removal efficiency of about 96% from 45 min to 120 min of the contact time. Accordingly, the NCC adsorption on the PVBTAC-modified *α*-Al_2_O_3_ surface reached an equilibrium state at the contact time of 45 min. Therefore, the optimal contact time of 45 min was applied for the next investigation.

#### 3.3.5. NCC Adsorption Isotherm Model

**Figure 10:**
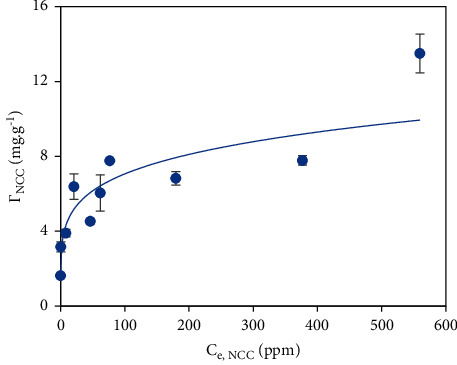
NCC adsorption on the PVBTAC-modified *α*-Al_2_O_3_ particles was well fitted with Freundlich model at conditions: *C*_*i*,NCC_ from 10 to 600 ppm, *pH* 8, NaCl 10 mM, *t* of 45 min, and *m*_*α*-_Al_2_O_3_ of 3 mg mL^−1^.

The fitness of the NCC adsorption on the PVBTAC-modified *α*-Al_2_O_3_ particles with Langmuir, Freundlich and two-step models was examined. It can be seen clearly in Figure 10 that *Г*_NCC_ gradually raised from about 1.62 to 13.5 mg g^−1^ as the NCC initial concentration increased from 5 to 600 ppm. A maximum NCC adsorption capacity of 13.5 mg g^−1^ determined proves that PVBTAC-modified *α*-Al_2_O_3_ was high-performance adsorbents in the NCC removal compared with other materials. The NCC adsorption capacity by using PVBTAC-modified *α*-Al_2_O_3_ particles was higher than using activated carbon as adsorbents (Table 1) [47–49]. Moreover, it might suggest that more numerous surface sites were available for NCC adsorption at low NCC initial concentration, intensifying the electrostatic attractions between NCC and adsorbed PVBTAC. Oppositely, the repulsive interactions between the NCC molecules were dominant at high NCC initial concentration. The NCC adsorptive removal only followed the Freundlich model with the fitting parameters calculated as *K*_*f*_ of 2.8341 and *n* of 0.1983 and not adapted with the Langmuir and two-step models (Figure 10).

#### 3.3.6. NCC Adsorption Kinetics on PVBTAC-Modified *α*-Al_2_O_3_ Particles

**Figure 11:**
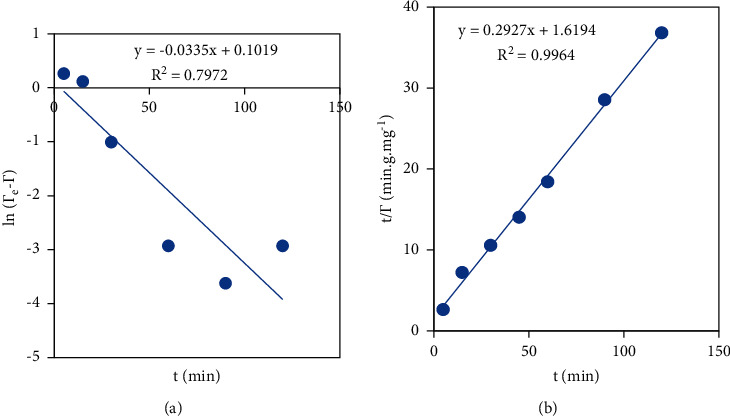
Kinetics of the PVBTAC adsorption isotherm on the *α*-Al_2_O_3_ particles following (a) pseudo-first and (b) pseudo-second at conditions of *C*_*i*, NCC_ of 10 ppm, pH 8, NaCl 10 mM, and *m*_*α*-Al2O3_ of 3 mg mL^−1^.

Two kinetic models including pseudo-first and pseudo-second were considered for the NCC adsorption on PVBTAC-modified *α*-Al_2_O_3_ particles, as indicated in Figure 11.

The NCC adsorption kinetics on the PVBTAC-modified *α*-Al_2_O_3_ surface were better followed with the pseudo-second model with a higher correlation coefficient (*R*^2^) of 0.9964 than the pseudo-first model with a lower *R*^2^ of 0.7972 (Figure 11). It was proposed that there were some interactions between the adsorbed NCC molecules. In detail, the interactions might be resulted from hydrogen bondings between −OH groups in the NCC suggested by the FT-IR spectra above. The fitted parameters were shown in Table 2.

#### 3.3.7. Regeneration of *α*-Al_2_O_3_ Adsorbent

**Figure 12:**
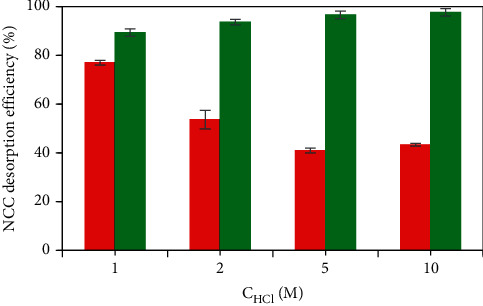
Effect of the HCl concentration to regenerate *α*-Al_2_O_3_ adsorbent with different HCl treatment times: (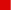) 1^st^ and (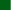) 2^nd^.

Formerly, acidic solutions of 1, 2, 5 and 10 M HCl were used to regenerate *α*-Al_2_O_3_ adsorbent after the NCC adsorption. The regeneration procedure of the *α*-Al_2_O_3_ adsorbent was conducted twice. For each time, the PVBTAC-modified *α*-Al_2_O_3_ particles adsorbed the NCC were shaken with desired HCl concentration for 45 min. Then the concentration of the NCC desorbed was determined by the UV-Vis method at the wavelength of 508 nm. As shown in Figure 12, the NCC desorption efficiency in the first HCl treatment was low. After HCl treatment twice, the NCC adsorption efficiency reached higher than 90% at all HCl concentration. Moreover, the NCC desorption efficiency slightly raised with increasing 2-fold HCl concentration from 1 to 2 M, and reached maximum (approximately 97%) at the HCl higher than 5 M. It was referred to elute almost the NCC from the adsorbent surface. More presence of OH_2_^+^ groups on the particle surface under acidic condition led to stronger electrostatic repulsion between the positively charged PVBTAC and the positively charged *α*-Al_2_O_3_. As a result, the NCC were desorbed. Therefore, the regeneration of the *α*-Al_2_O_3_ adsorbent was carried out twice by using the HCl solution of 5 M. Each procedure of the HCl treatment twice was considered to be a regeneration time. The regeneration treatment of the *α*-Al_2_O_3_ adsorbents with strong acid at high concentration confirmed strong interactions between NCC and PVBTAC-modified surface, and the high synthesized adsorbent stability.

**Figure 13:**
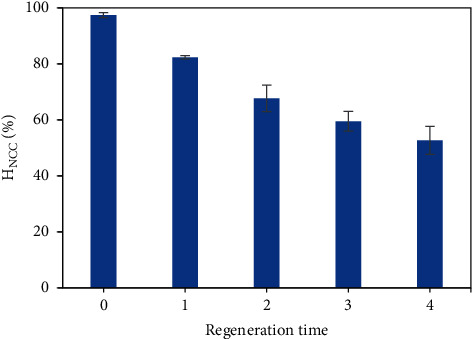
The NCC removal efficiency by adsorbing onto the PVBTAC-modified-regenerated *α*-Al_2_O_3_ adsorbents with different regeneration times at optimal conditions of: *C*_*i*,NCC_ of 10 ppm, *pH* 8, NaCl 10 mM, and *m*_*α*-Al2O3_ of 3 mg mL^−1^.

Lately, the regenerated *α*-Al_2_O_3_ particles were modified by the PVBTAC adsorption. Then the PVBTAC-modified-regenerated adsorbents were applied to adsorptively remove NCC. It was seen in Figure 13 that the NCC removal efficiency by using PVBTAC-modified-regenerated adsorbents decreased with rising the regeneration time. However, the NCC removal efficiency, achieved about 53% after four regeneration times, still was high. It was clarified that high reusability of the *α*-Al_2_O_3_ adsorbents.

## 4. Conclusions

In the present study, it was the first time the azo dye NCC was highly adsorptively removed from aqueous solutions by using the PVBTAC polycation-modified *α*-Al_2_O_3_ particles. Both PVBTAC adsorption and NCC adsorption were comprehensively investigated. To sufficiently modify the *α*-Al_2_O_3_ particles, polycations PVBTAC of 1000 ppm initial concentration were adsorbed onto *α*-Al_2_O_3_ for 2 h at *pH* 8 under ionic strength of NaCl 100 mM. Then, the NCC adsorption on the PVBTAC-modified *α*-Al_2_O_3_ particles was optimized at conditions including *pH* 8, NaCl 10 mM, contact time of 45 min, and an *α*-Al_2_O_3_ dosage of 3 mg g^−1^. The adsorption of both PVBTAC and NCC was controlled by the electrostatic and non-interaction that also affirmed by the FT-IR spectra. The mechanism and kinetics of the NCC adsorption isotherm onto the PVBTAC-modified *α*-Al_2_O_3_ particles were clarified. The NCC adsorption isotherm mechanism was in accordance with the Freundlich model, while the NCC adsorption kinetics was more suitably followed by the pseudo-second than the pseudo-first model. The nanosized alpha alumina was proved to be a highly reusable adsorbent.

## Figures and Tables

**Table 1:** NCC adsorption capacities and NCC removal efficiencies by using different adsorbents.

Adsorbents	Adsorption capacity (mg g^−1^)	Removal efficiency (%)	References
PVBTAC-modified *α*-Al_2_O_3_	13.5	97.5	This study
Chemically treated mangrove barks	12.72	25.40	[47]
Activated carbon prepared from poplar woods	3.91	31.28	[48]
Activated carbon prepared from almond shells	10.75	90.83	[49]

**Table 2:** Fitting parameters for the NCC adsorption onto the PVBTAC-modified *α*-Al_2_O_3_ particles following the pseudo-first and pseudo-second kinetic.

Parameters	*Г*_e_ (mg.g^−1^)	*R* ^2^	*K*_1_ (min^−1^)	*K*_2_ (g mg^−1^ min^−1^)
Pseudo-first kinetic	3.21	0.7972	0.0335	
Pseudo-second kinetic	3.21	0.9964	-	0.0600

## Data Availability

All the data and supporting materials are included within the article.
